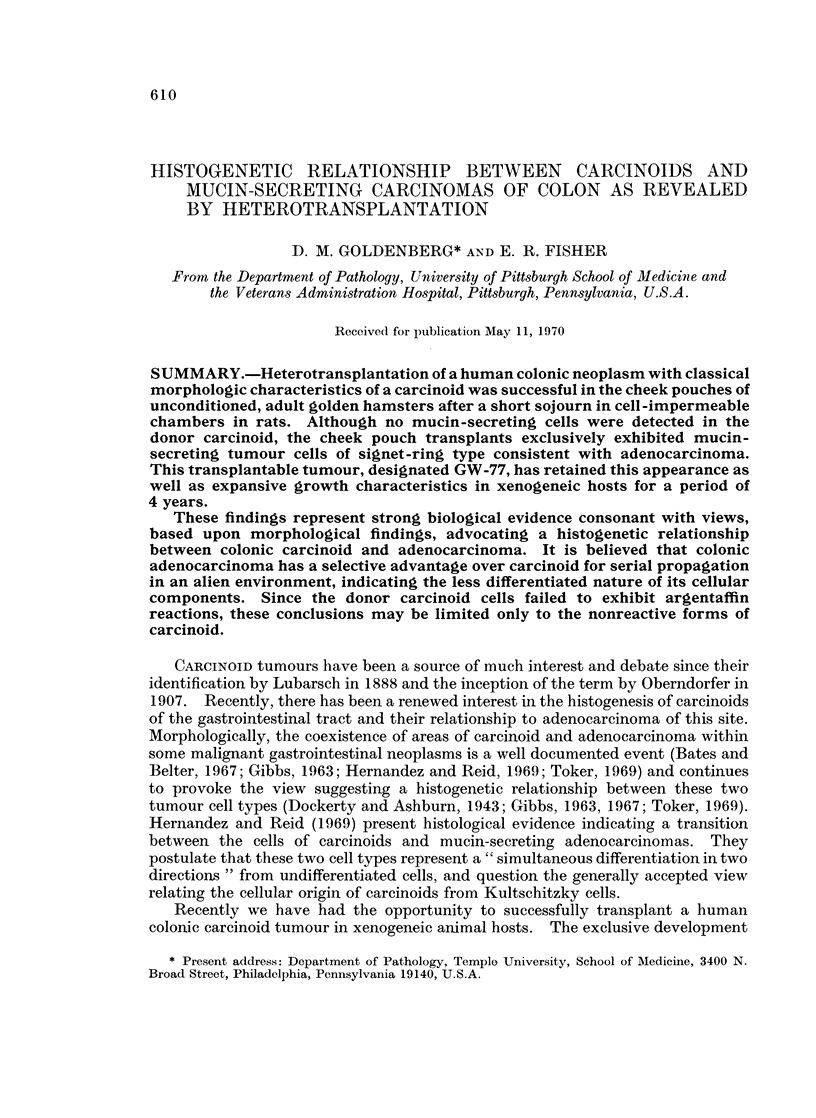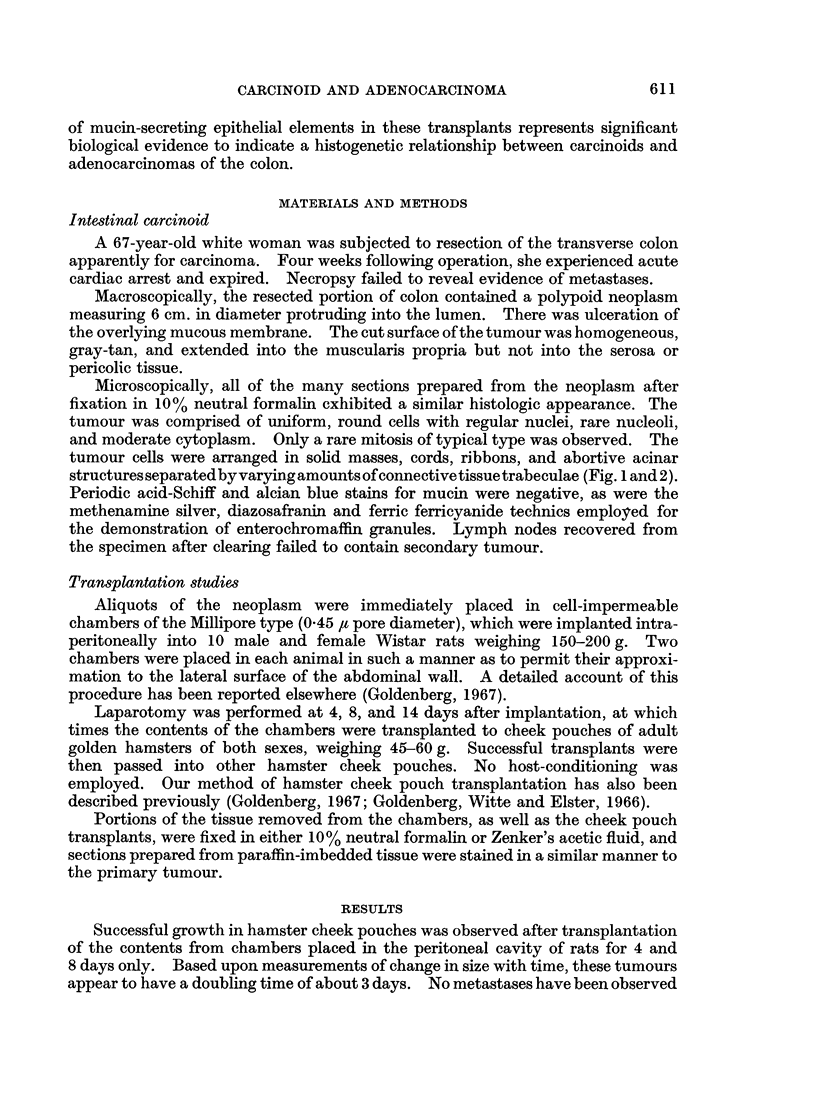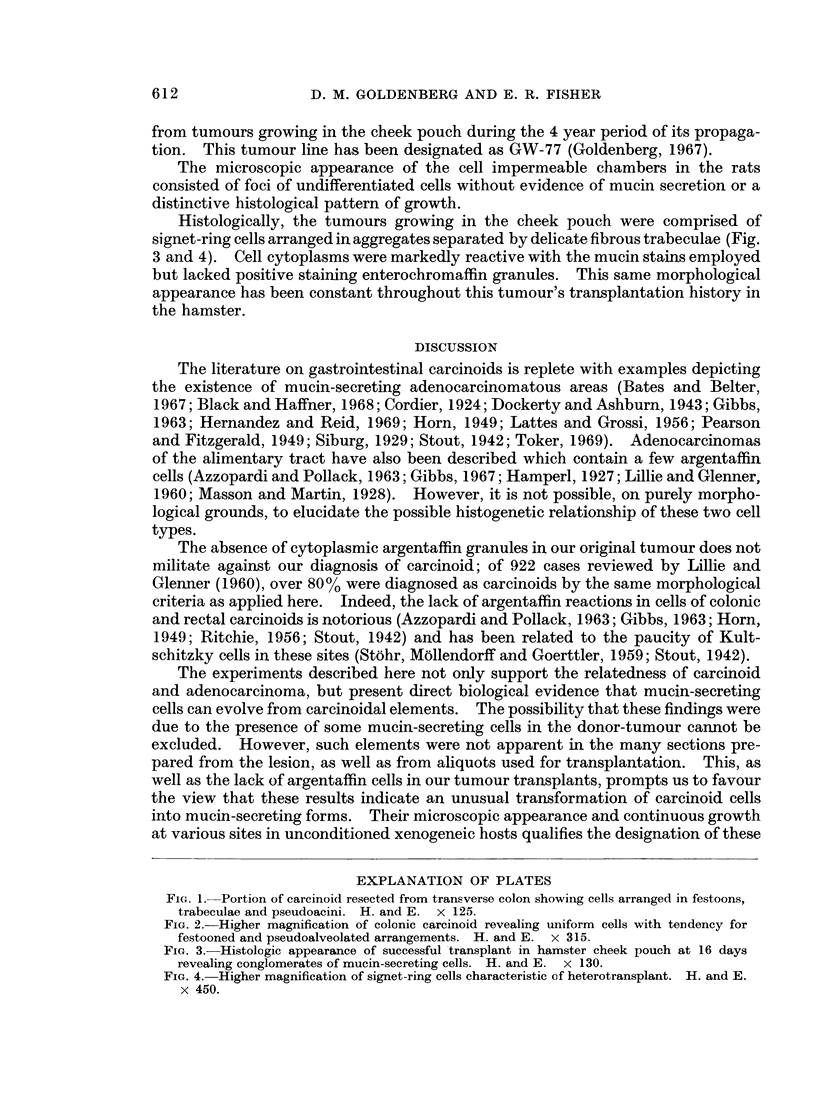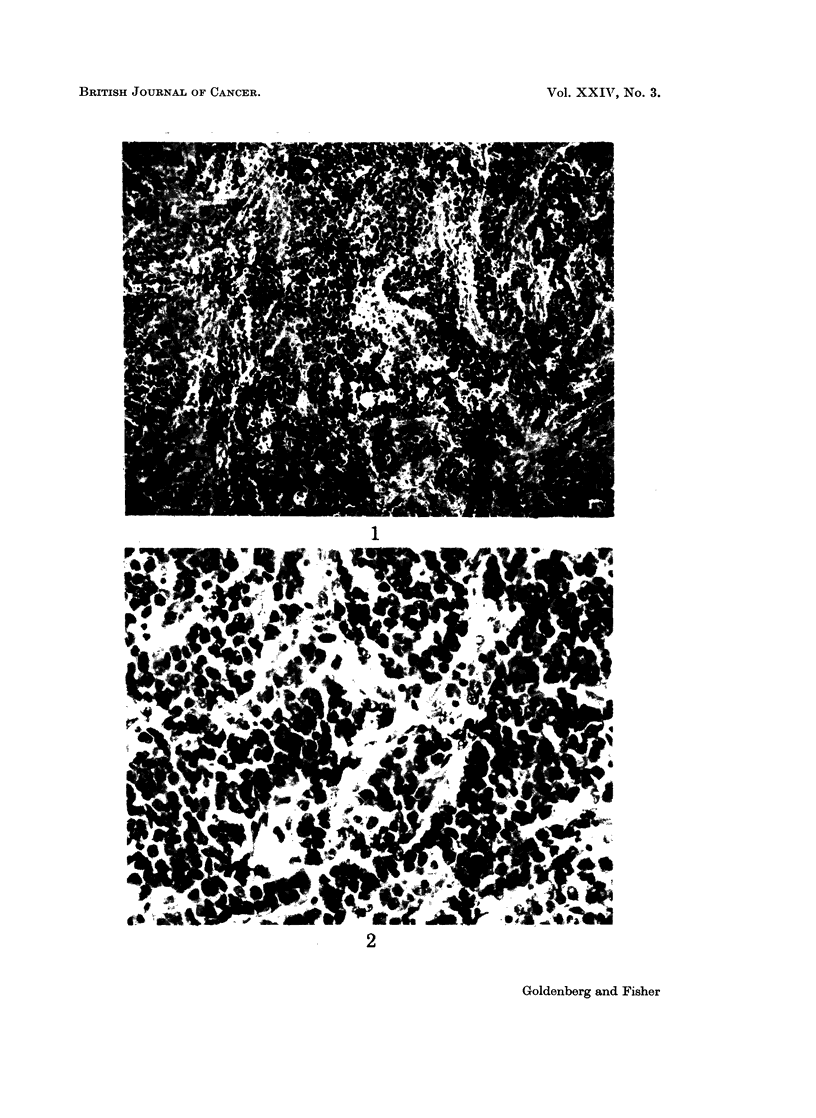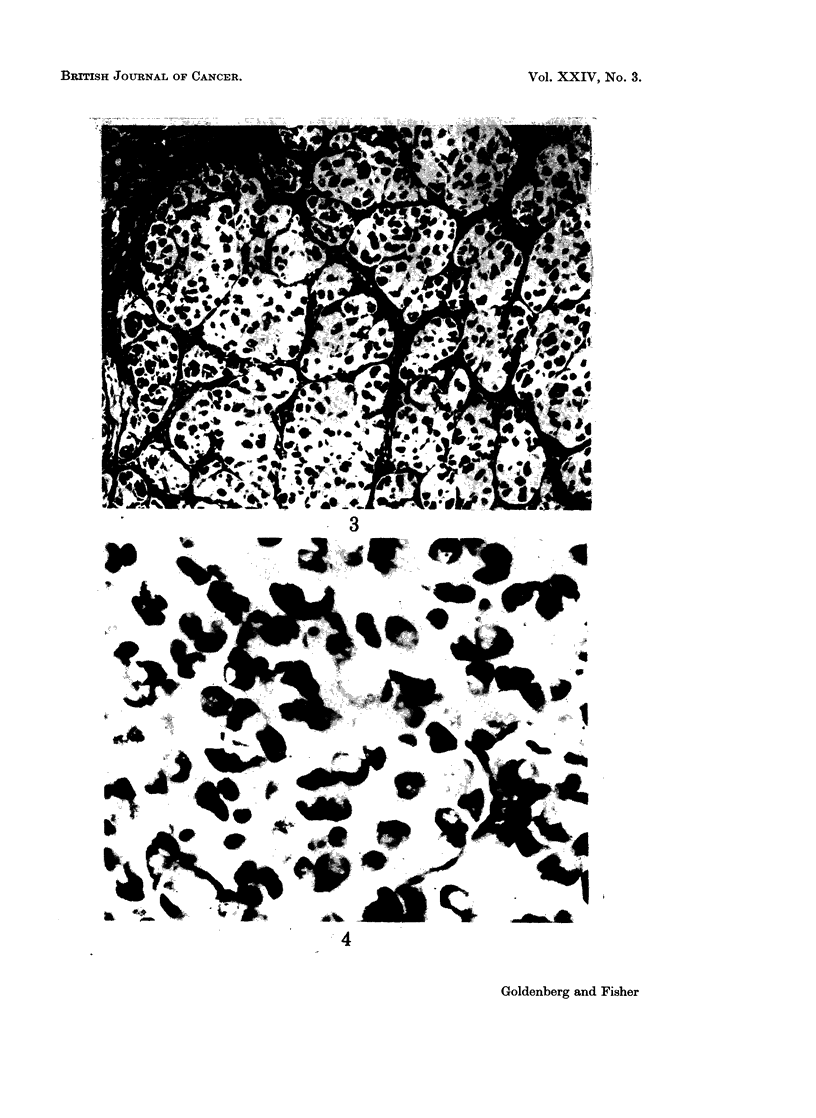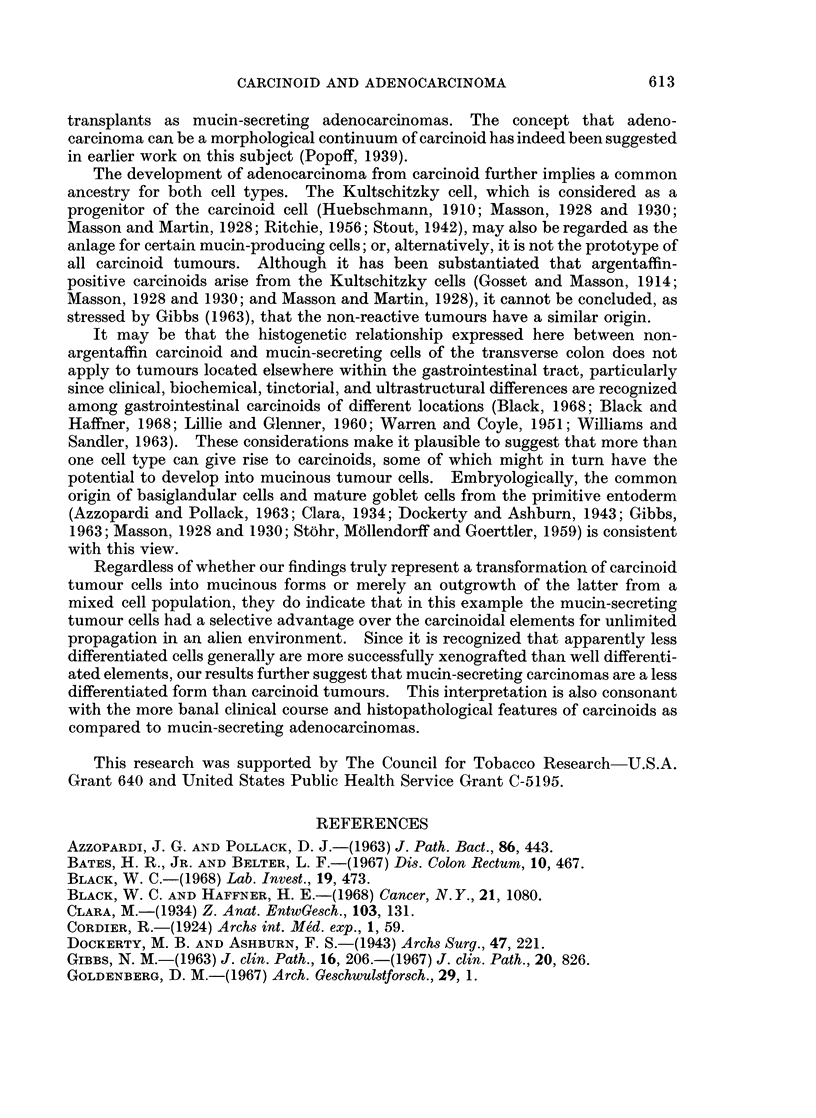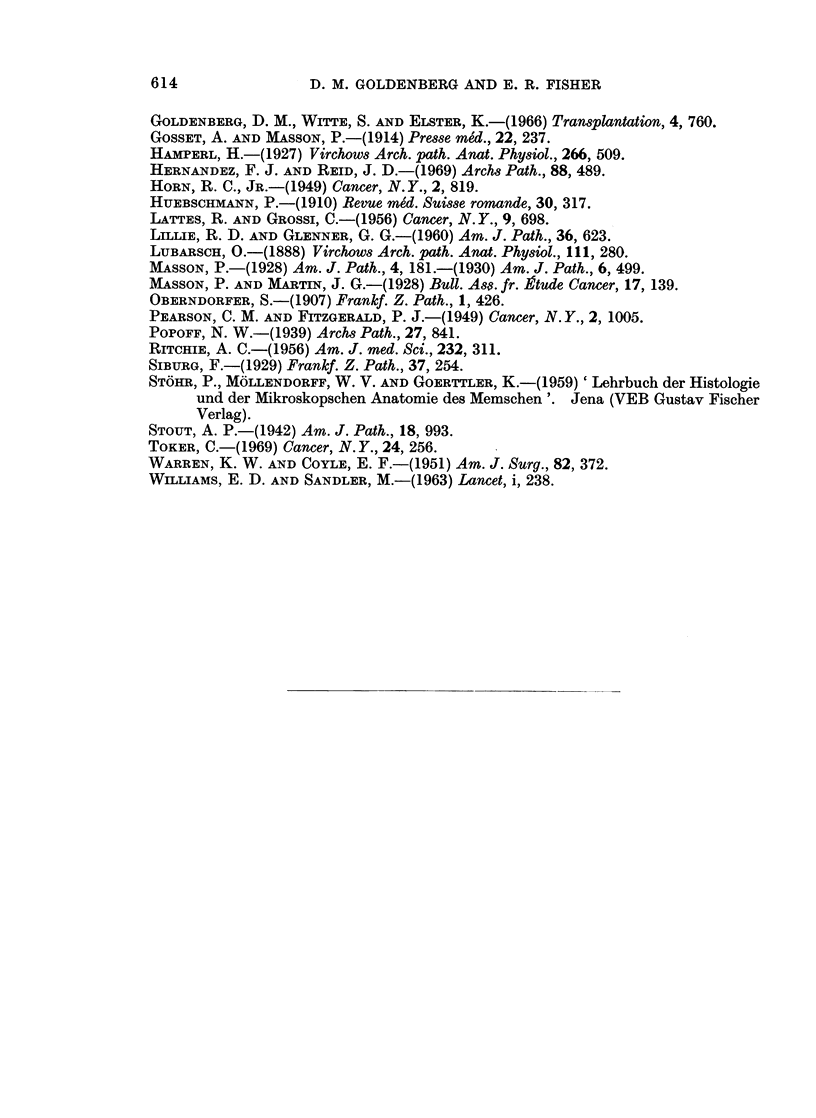# Histogenetic Relationship Between Carcinoids and Mucin-secreting Carcinomas of Colon as Revealed by Heterotransplantation

**DOI:** 10.1038/bjc.1970.74

**Published:** 1970-09

**Authors:** D. M. Goldenberg, E. R. Fisher

## Abstract

**Images:**


### HISTOGENETIC RELATIONSHIP BETWEEN CARCINOIDS AND MUCIN-SECRETING CARCINOMAS OF COLON AS REVEALED BY HETEROTRANSPLANTATION

D. M. GOLDENBERG* AND E. R. FISHER

*From the Department of Pathology, University of Pittsburgh School of Medicine and the Veterans Administration Hospital, Pittsburgh, Pennsylvania, U.S.A.*

Received for publication May 11, 1970

**SUMMARY.—Heterotransplantation of a human colonic neoplasm with classical morphologic characteristics of a carcinoid was successful in the cheek pouches of unconditioned, adult golden hamsters after a short sojourn in cell-impermeable chambers in rats. Although no mucin-secreting cells were detected in the donor carcinoid, the cheek pouch transplants exclusively exhibited mucin-secreting tumour cells of signet-ring type consistent with adenocarcinoma. This transplantable tumour, designated GW-77, has retained this appearance as well as expansive growth characteristics in xenogeneic hosts for a period of 4 years.**

**These findings represent strong biological evidence consonant with views, based upon morphological findings, advocating a histogenetic relationship between colonic carcinoid and adenocarcinoma. It is believed that colonic adenocarcinoma has a selective advantage over carcinoid for serial propagation in an alien environment, indicating the less differentiated nature of its cellular components. Since the donor carcinoid cells failed to exhibit argentaffin reactions, these conclusions may be limited only to the nonreactive forms of carcinoid.**

CARCINOID tumours have been a source of much interest and debate since their identification by Lubarsch in 1888 and the inception of the term by Oberndorfer in 1907. Recently, there has been a renewed interest in the histogenesis of carcinoids of the gastrointestinal tract and their relationship to adenocarcinoma of this site. Morphologically, the coexistence of areas of carcinoid and adenocarcinoma within some malignant gastrointestinal neoplasms is a well documented event (Bates and Belter, 1967; Gibbs, 1963; Hernandez and Reid, 1969; Toker, 1969) and continues to provoke the view suggesting a histogenetic relationship between these two tumour cell types (Dockerty and Ashburn, 1943; Gibbs, 1963, 1967; Toker, 1969). Hernandez and Reid (1969) present histological evidence indicating a transition between the cells of carcinoids and mucin-secreting adenocarcinomas. They postulate that these two cell types represent a " simultaneous differentiation in two directions " from undifferentiated cells, and question the generally accepted view relating the cellular origin of carcinoids from Kultschitzky cells.

Recently we have had the opportunity to successfully transplant a human colonic carcinoid tumour in xenogeneic animal hosts. The exclusive development

* Present address: Department of Pathology, Temple University, School of Medicine, 3400 N. Broad Street, Philadelphia, Pennsylvania 19140, U.S.A.

of mucin-secreting epithelial elements in these transplants represents significant biological evidence to indicate a histogenetic relationship between carcinoids and adenocarcinomas of the colon.

### MATERIALS AND METHODS

### *Intestinal carcinoid*

A 67-year-old white woman was subjected to resection of the transverse colon apparently for carcinoma. Four weeks following operation, she experienced acute cardiac arrest and expired. Necropsy failed to reveal evidence of metastases.

Macroscopically, the resected portion of colon contained a polypoid neoplasm measuring 6 cm. in diameter protruding into the lumen. There was ulceration of the overlying mucous membrane. The cut surface of the tumour was homogeneous, gray-tan, and extended into the muscularis propria but not into the serosa or pericolic tissue.

Microscopically, all of the many sections prepared from the neoplasm after fixation in 10% neutral formalin cxhibited a similar histologic appearance. The tumour was comprised of uniform, round cells with regular nuclei, rare nucleoli, and moderate cytoplasm. Only a rare mitosis of typical type was observed. The tumour cells were arranged in solid masses, cords, ribbons, and abortive acinar structures separated by varying amounts of connective tissue trabeculae (Fig. 1 and 2). Periodic acid-Schiff and alcian blue stains for mucin were negative, as were the methenamine silver, diazosafranin and ferric ferricyanide technics employed for the demonstration of enterochromaffin granules. Lymph nodes recovered from the specimen after clearing failed to contain secondary tumour.

### *Transplantation studies*

Aliquots of the neoplasm were immediately placed in cell-impermeable chambers of the Millipore type (0·45 $\mu$ pore diameter), which were implanted intraperitoneally into 10 male and female Wistar rats weighing 150–200 g. Two chambers were placed in each animal in such a manner as to permit their approximation to the lateral surface of the abdominal wall. A detailed account of this procedure has been reported elsewhere (Goldenberg, 1967).

Laparotomy was performed at 4, 8, and 14 days after implantation, at which times the contents of the chambers were transplanted to cheek pouches of adult golden hamsters of both sexes, weighing 45–60 g. Successful transplants were then passed into other hamster cheek pouches. No host-conditioning was employed. Our method of hamster cheek pouch transplantation has also been described previously (Goldenberg, 1967; Goldenberg, Witte and Elster, 1966).

Portions of the tissue removed from the chambers, as well as the cheek pouch transplants, were fixed in either 10% neutral formalin or Zenker's acetic fluid, and sections prepared from paraffin-imbedded tissue were stained in a similar manner to the primary tumour.

### RESULTS

Successful growth in hamster cheek pouches was observed after transplantation of the contents from chambers placed in the peritoneal cavity of rats for 4 and 8 days only. Based upon measurements of change in size with time, these tumours appear to have a doubling time of about 3 days. No metastases have been observed from tumours growing in the cheek pouch during the 4 year period of its propagation. This tumour line has been designated as GW-77 (Goldenberg, 1967).

The microscopic appearance of the cell impermeable chambers in the rats consisted of foci of undifferentiated cells without evidence of mucin secretion or a distinctive histological pattern of growth.

Histologically, the tumours growing in the cheek pouch were comprised of signet-ring cells arranged in aggregates separated by delicate fibrous trabeculae (Fig. 3 and 4). Cell cytoplasms were markedly reactive with the mucin stains employed but lacked positive staining enterochromaffin granules. This same morphological appearance has been constant throughout this tumour's transplantation history in the hamster.

### DISCUSSION

The literature on gastrointestinal carcinoids is replete with examples depicting the existence of mucin-secreting adenocarcinomatous areas (Bates and Belter, 1967; Black and Haffner, 1968; Cordier, 1924; Dockerty and Ashburn, 1943; Gibbs, 1963; Hernandez and Reid, 1969; Horn, 1949; Lattes and Grossi, 1956; Pearson and Fitzgerald, 1949; Siburg, 1929; Stout, 1942; Toker, 1969). Adenocarcinomas of the alimentary tract have also been described which contain a few argentaffin cells (Azzopardi and Pollack, 1963; Gibbs, 1967; Hamperl, 1927; Lillie and Glenner, 1960; Masson and Martin, 1928). However, it is not possible, on purely morphological grounds, to elucidate the possible histogenetic relationship of these two cell types.

The absence of cytoplasmic argentaffin granules in our original tumour does not militate against our diagnosis of carcinoid; of 922 cases reviewed by Lillie and Glenner (1960), over 80% were diagnosed as carcinoids by the same morphological criteria as applied here. Indeed, the lack of argentaffin reactions in cells of colonic and rectal carcinoids is notorious (Azzopardi and Pollack, 1963; Gibbs, 1963; Horn, 1949; Ritchie, 1956; Stout, 1942) and has been related to the paucity of Kultschitzky cells in these sites (Stöhr, Möllendorff and Goerttler, 1959; Stout, 1942).

The experiments described here not only support the relatedness of carcinoid and adenocarcinoma, but present direct biological evidence that mucin-secreting cells can evolve from carcinoidal elements. The possibility that these findings were due to the presence of some mucin-secreting cells in the donor-tumour cannot be excluded. However, such elements were not apparent in the many sections prepared from the lesion, as well as from aliquots used for transplantation. This, as well as the lack of argentaffin cells in our tumour transplants, prompts us to favour the view that these results indicate an unusual transformation of carcinoid cells into mucin-secreting forms. Their microscopic appearance and continuous growth at various sites in unconditioned xenogeneic hosts qualifies the designation of these

---

### EXPLANATION OF PLATES

FIG. 1.—Portion of carcinoid resected from transverse colon showing cells arranged in festoons, trabeculae and pseudoacini. H. and E. × 125.

FIG. 2.—Higher magnification of colonic carcinoid revealing uniform cells with tendency for festooned and pseudoalveolated arrangements. H. and E. × 315.

FIG. 3.—Histologic appearance of successful transplant in hamster cheek pouch at 16 days revealing conglomerates of mucin-secreting cells. H. and E. × 130.

FIG. 4.—Higher magnification of signet-ring cells characteristic of heterotransplant. H. and E. × 450.

1

2

Goldenberg and Fisher

3

4

Goldenberg and Fisher transplants as mucin-secreting adenocarcinomas. The concept that adenocarcinoma can be a morphological continuum of carcinoid has indeed been suggested in earlier work on this subject (Popoff, 1939).

The development of adenocarcinoma from carcinoid further implies a common ancestry for both cell types. The Kultschitzky cell, which is considered as a progenitor of the carcinoid cell (Huebschmann, 1910; Masson, 1928 and 1930; Masson and Martin, 1928; Ritchie, 1956; Stout, 1942), may also be regarded as the anlage for certain mucin-producing cells; or, alternatively, it is not the prototype of all carcinoid tumours. Although it has been substantiated that argentaffin-positive carcinoids arise from the Kultschitzky cells (Gosset and Masson, 1914; Masson, 1928 and 1930; and Masson and Martin, 1928), it cannot be concluded, as stressed by Gibbs (1963), that the non-reactive tumours have a similar origin.

It may be that the histogenetic relationship expressed here between non-argentaffin carcinoid and mucin-secreting cells of the transverse colon does not apply to tumours located elsewhere within the gastrointestinal tract, particularly since clinical, biochemical, tinctorial, and ultrastructural differences are recognized among gastrointestinal carcinoids of different locations (Black, 1968; Black and Haffner, 1968; Lillie and Glenner, 1960; Warren and Coyle, 1951; Williams and Sandler, 1963). These considerations make it plausible to suggest that more than one cell type can give rise to carcinoids, some of which might in turn have the potential to develop into mucinous tumour cells. Embryologically, the common origin of basiglandular cells and mature goblet cells from the primitive entoderm (Azzopardi and Pollack, 1963; Clara, 1934; Dockerty and Ashburn, 1943; Gibbs, 1963; Masson, 1928 and 1930; Stöhr, Möllendorff and Goerttler, 1959) is consistent with this view.

Regardless of whether our findings truly represent a transformation of carcinoid tumour cells into mucinous forms or merely an outgrowth of the latter from a mixed cell population, they do indicate that in this example the mucin-secreting tumour cells had a selective advantage over the carcinoidal elements for unlimited propagation in an alien environment. Since it is recognized that apparently less differentiated cells generally are more successfully xenografted than well differentiated elements, our results further suggest that mucin-secreting carcinomas are a less differentiated form than carcinoid tumours. This interpretation is also consonant with the more banal clinical course and histopathological features of carcinoids as compared to mucin-secreting adenocarcinomas.

This research was supported by The Council for Tobacco Research—U.S.A. Grant 640 and United States Public Health Service Grant C-5195.

### REFERENCES

Azzopardi, J. G. and Pollack, D. J.—(1963) *J. Path. Bact.*, **86**, 443.
Bates, H. R., Jr. and Belter, L. F.—(1967) *Dis. Colon Rectum*, **10**, 467.
Black, W. C.—(1968) *Lab. Invest.*, **19**, 473.
Black, W. C. and Haffner, H. E.—(1968) *Cancer, N.Y.*, **21**, 1080.
Clara, M.—(1934) *Z. Anat. EntwGesch.*, **103**, 131.
Cordier, R.—(1924) *Archs int. Méd. exp.*, **1**, 59.
Dockerty, M. B. and Ashburn, F. S.—(1943) *Archs Surg.*, **47**, 221.
Gibbs, N. M.—(1963) *J. clin. Path.*, **16**, 206.—(1967) *J. clin. Path.*, **20**, 826.
Goldenberg, D. M.—(1967) *Arch. Geschwulstforsch.*, **29**, 1.

GOLDENBERG, D. M., WITTE, S. AND ELSTER, K.—(1966) *Transplantation*, **4**, 760.
GOSSET, A. AND MASSON, P.—(1914) *Presse méd.*, **22**, 237.
HAMPERL, H.—(1927) *Virchows Arch. path. Anat. Physiol.*, **266**, 509.
HERNANDEZ, F. J. AND REID, J. D.—(1969) *Archs Path.*, **88**, 489.
HORN, R. C., JR.—(1949) *Cancer, N.Y.*, **2**, 819.
HUEBSCHMANN, P.—(1910) *Revue méd. Suisse romande*, **30**, 317.
LATTES, R. AND GROSSI, C.—(1956) *Cancer, N.Y.*, **9**, 698.
LILLIE, R. D. AND GLENNER, G. G.—(1960) *Am. J. Path.*, **36**, 623.
LUBARSCH, O.—(1888) *Virchows Arch. path. Anat. Physiol.*, **111**, 280.
MASSON, P.—(1928) *Am. J. Path.*, **4**, 181.—(1930) *Am. J. Path.*, **6**, 499.
MASSON, P. AND MARTIN, J. G.—(1928) *Bull. Ass. fr. Étude Cancer*, **17**, 139.
OBERNDORFER, S.—(1907) *Frankf. Z. Path.*, **1**, 426.
PEARSON, C. M. AND FITZGERALD, P. J.—(1949) *Cancer, N.Y.*, **2**, 1005.
POPOFF, N. W.—(1939) *Archs Path.*, **27**, 841.
RITCHIE, A. C.—(1956) *Am. J. med. Sci.*, **232**, 311.
SIBURG, F.—(1929) *Frankf. Z. Path.*, **37**, 254.
STÖHR, P., MÖLLENDORFF, W. V. AND GOERTTLER, K.—(1959) 'Lehrbuch der Histologie und der Mikroskopschen Anatomie des Memschen'. Jena (VEB Gustav Fischer Verlag).
STOUT, A. P.—(1942) *Am. J. Path.*, **18**, 993.
TOKER, C.—(1969) *Cancer, N.Y.*, **24**, 256.
WARREN, K. W. AND COYLE, E. F.—(1951) *Am. J. Surg.*, **82**, 372.
WILLIAMS, E. D. AND SANDLER, M.—(1963) *Lancet*, i, 238.